# Integrative analysis of the *Trypanosoma brucei* gene expression cascade predicts differential regulation of mRNA processing and unusual control of ribosomal protein expression

**DOI:** 10.1186/s12864-016-2624-3

**Published:** 2016-04-26

**Authors:** Enoch B. Antwi, Jurgen R. Haanstra, Gowthaman Ramasamy, Bryan Jensen, Dorothea Droll, Federico Rojas, Igor Minia, Monica Terrao, Clémentine Mercé, Keith Matthews, Peter J. Myler, Marilyn Parsons, Christine Clayton

**Affiliations:** Zentrum für Molekulare Biologie der Universität Heidelberg, DKFZ-ZMBH Alliance, Im Neuenheimer Feld 282, D-69120 Heidelberg, Germany; Department of Molecular Cell Physiology, Vrije Universiteit Amsterdam, De Boelelaan 1085, 1081 HV Amsterdam, The Netherlands; Systems Bioinformatics, Vrije Universiteit Amsterdam, De Boelelaan 1085, 1081 HV Amsterdam, The Netherlands; Center for Infectious Disease Research (formerly Seattle Biomedical Research Institute), 307 Westlake Ave N, Seattle, WA 98109-5219 USA; Centre for Immunity, Infection and Evolution, Institute for Immunology and infection Research, School of Biological Sciences, Ashworth Laboratories, University of Edinburgh, West Mains Road, Edinburgh, EH9 3JT UK; Department of Global Health, University of Washington, Harris Hydraulics Building, 1705 NE Pacific St #310E, Box 357965, Seattle, WA 98195 USA; Department of Biomedical Informatics and Medical Education, University of Washington, Seattle, WA 98195 USA; Current address: Biology of Host Parasite Interactions, 25 rue du Docteur Roux, 75724 Paris cedex 15, France

## Abstract

**Background:**

*Trypanosoma brucei* is a unicellular parasite which multiplies in mammals (bloodstream form) and Tsetse flies (procyclic form). Trypanosome RNA polymerase II transcription is polycistronic, individual mRNAs being excised by *trans* splicing and polyadenylation. We previously made detailed measurements of mRNA half-lives in bloodstream and procyclic forms, and developed a mathematical model of gene expression for bloodstream forms. At the whole transcriptome level, many bloodstream-form mRNAs were less abundant than was predicted by the model.

**Results:**

We refined the published mathematical model and extended it to the procyclic form. We used the model, together with known mRNA half-lives, to predict the abundances of individual mRNAs, assuming rapid, unregulated mRNA processing; then we compared the results with measured mRNA abundances. Remarkably, the abundances of most mRNAs in procyclic forms are predicted quite well by the model, being largely explained by variations in mRNA decay rates and length. In bloodstream forms substantially more mRNAs are less abundant than predicted. We list mRNAs that are likely to show particularly slow or inefficient processing, either in both forms or with developmental regulation. We also measured ribosome occupancies of all mRNAs in trypanosomes grown in the same conditions as were used to measure mRNA turnover. In procyclic forms there was a weak positive correlation between ribosome density and mRNA half-life, suggesting cross-talk between translation and mRNA decay; ribosome density was related to the proportion of the mRNA on polysomes, indicating control of translation initiation. Ribosomal protein mRNAs in procyclics appeared to be exceptionally rapidly processed but poorly translated.

**Conclusions:**

Levels of mRNAs in procyclic form trypanosomes are determined mainly by length and mRNA decay, with some control of precursor processing. In bloodstream forms variations in nuclear events play a larger role in transcriptome regulation, suggesting aquisition of new control mechanisms during adaptation to mammalian parasitism.

**Electronic supplementary material:**

The online version of this article (doi:10.1186/s12864-016-2624-3) contains supplementary material, which is available to authorized users.

## Background

Organisms of the family Kinetoplastidea are unicellar flagellated eukaryotes which can be free-living or parasitic. One of the most remarkable features of Kinetoplastid molecular biology is the arrangement of protein-coding genes in polycistronic transcription units [1–4], which precludes control of transcription at the level of individual mRNAs. All evidence obtained to date indicates that trancription initiation is determined by chromatin modification [5]. During exponential growth, differences in initiation between the different polymerase II transcription units have not been observed. The only documented polymerase II control is global down-regulation of initiation upon heat shock or the attainment of stationary phase; this impacts open reading frames at the beginning of transcription units earlier than those at the ends [6]. The lack of transcriptional control at the level of individual genes means that differences in steady-state gene expression must be effected post-transcriptionally. The most extensive analyses of gene expression have been for the African trypanosome *Trypanosoma brucei*, which is the subject of this paper. Nearly all studies of in vitro cultured *T. brucei* have concentrated on the bloodstream form (similar to the form that multiplies in mammalian blood) and the procyclic form (similar to the form that multiplies in the midgut of Tsetse flies). In *T. brucei*, the transcription units that include the genes encoding major surface proteins of the bloodstream and procyclic forms - variant surface glycoproteins and the procyclins - are important exceptions to the general rule: they are transcribed in a developmentally-regulated fashion by RNA polymerase I [7].

**Fig. 1:**
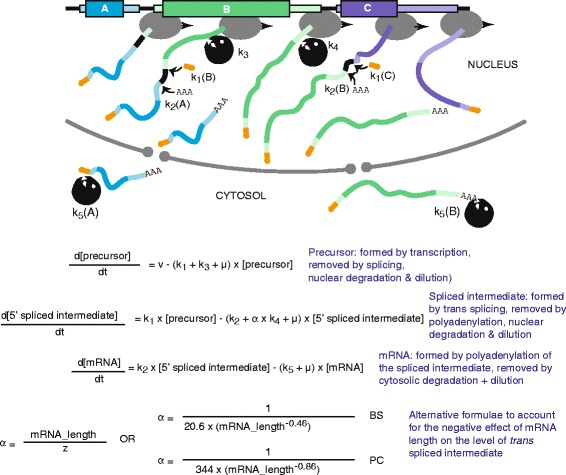
Gene expression from DNA to mRNA in *Trypanosoma brucei*. The upper panel is a schematic time-lapse image of a polymerase II complex progressing along a chromosome. Coding regions are in dark colours and 3′ and 5′ untranslated regions are in lighter colours. The capped spliced leader is in orange. Kinetic constants for the different processes are indicated and the formulae that comprise the model are shown below the figure 5′ *trans* splicing (rate constant k_1_) and 3′ polyadenylation (rate constant k_2_) compete with nuclear degradation of the precursor (rate constant k_3_) and the 5′ spliced intermediate (rate constant k_4_). Assuming that the two processes are coupled, the rate constant for 3′ polyadenylation (k_2_) of mRNA A is expected to equal the rate constant for 5′ *trans* splicing (k_1_) of mRNA B, and the same applies for mRNA and mRNA C (k_2_ of mRNA B = k_1_ for mRNA C). Based on the observation that that long mRNAs had unexpectedly low abundances, we added a factor (α) that incorporates length-dependent precursor degradation into the model [16]. In the analysis described in this paper, we tested alternative versions of this and used a different factor for PC trypanosomes (see text and Table 1). Finally, there is degradation of the mature mRNA (rate constant k_5_). Values for k_5_ are based on transcriptome-wide decay measurements. In addition, the growth rate of the cells is included via the specific growth rate μ. The growth rate affects the abundance of every RNA species, as during growth the pre-existing RNA species get diluted

Individual Kinetoplastid mRNAs are generated from the precursor by processing (Fig. 1). The capped 5′ end is generated by *trans* splicing of a 39 nt, capped “spliced leader” (*SL*) [8]. Canonical *trans* splicing signals consist of a polypyrimidine tract followed by an AG dinucleotide [9, 10]. Use of splice sites of varying efficiencies - and choices between alternative sites - are thus the first potential point for post-transcriptional regulation [8]. The polyadenylation of an mRNA is temporally and mechanistically linked to downstream *trans* splicing, and thus depends on recognition of the downstream *trans* splicing signal by the splicing machinery [11–15]. In some cases, this downstream *trans* splicing event creates another protein-coding mRNA; in others, the splicing can form a non-coding RNA that is subsequently destroyed. For many mRNAs, both alternatives are possible, due to the presence of more than one splicing signal in the region preceding the next gene downstream. At steady state and with constant transcription, the processing rate by itself *cannot* affect the amount of mature mRNA that is made: no matter how long it takes, the mRNA will be made eventually [16]. In fact, though, the precursor is subject to degradation [17]. So if processing is slow, the precursor may be destroyed before it can be processed. Thus processing rates can indeed affect the amount of mRNA that is made. However, we currently do not know the extent to which splicing and polyadenylation rates contribute to mRNA levels.

Mature mRNAs are exported to the cytosol, where they are translated and/or degraded (Fig. 1). There is much more quantitative information available about these cytosolic events than there is about events in the nucleus. Numerous studies have documented differences in the decay rates of individual mRNAs both at steady state, and between different life-cycle stages [18]. The “Silicotryp” project aimed to quantify many aspects of trypanosome gene expression and metabolism, and to develop mathematical models that reflected the biology of the organism [19]. In the context of that project, using high-throughput cDNA sequencing (RNA-Seq), we completed transcriptome-wide measurements of mRNA decay rates in cultured bloodstream and procyclic forms [16]. Half-lives varied from less than 5 min to more than 4 h (beyond the capacity of the assay), with medians of 12 min for bloodstream forms and 20 min for procyclic forms. Levels of individual mRNAs varied from undetectable to over 100 mRNAs/cell/gene, with medians of 1.1 (procyclic forms) and 1.5 (bloodstream forms) [16]. We and others also measured ribosome occupancy of mRNA by ribosome profiling, finding a range from tightest possible packing to less than one ribosome per mRNA [20, 21]. The results thus indicate pervasive regulation of both mRNA decay and translation.

Mathematical modelling of gene expression can help us to understand how the kinetics of different steps influence mRNA levels [22–24]. Our first model of *T. brucei* gene expression was made for the mRNAs encoding phosphoglycerate kinase isoforms [17]. The model consists of a series of differential equations that describe transcription, mRNA *trans* splicing, and degradation of both the mRNA precursor and the mature mRNA (Fig. 1). For phosphoglycerate kinase, all parameters except the transcription rate were measured; the model then enabled us to estimate the transcription rate for this polycistronic unit [17]. The availability of accurate transcriptome-wide mRNA abundance and decay measurements [16] meant that we could test whether the model that had been developed for phosphoglycerate kinase could be applied transcriptome-wide. To do this, we simulated ‘virtual’ transcripts, rather than doing one-to-one comparisons with real individual mRNAs [16]. The cell division time is known, and we assumed that all genes are transcribed at the same rate, as estimated from the phosphoglycerate kinase example. The major unknown parameter was mRNA processing: for the sake of modelling, we therefore allowed this rate to vary within narrowly-defined limits, determined by a very small number of published individual measurements. Our initial analyses revealed that our model gave a distribution that was very similar to that measured for short mRNAs, but longer mRNAs had lower steady-state abundances than was predicted based on their half-lives. We therefore included length-dependent co-transcriptional degradation of precursor RNAs in the model [16] (Fig. 1). This is consistent with the idea that the longer the mRNA spends as a precursor, the more likely it is to be subjected to nuclear precursor degradation. The length correction considerably improved the fit of the model to the data.

A major use of mathematical models is to identify the existence of unknown factors or parameters. The extent to which a model fits the data depends on (a) the accuracy of the model and (b) the accuracy of the data that has been fed into the model. In our case, one of the most obvious data deficiencies was the absence, for almost all genes, of information concerning splicing and polyadenylation kinetics. We therefore set out to use the model to identify mRNAs which showed unusually slow, or stage-regulated, splicing. To do this, we first refined the model for bloodstream trypanosomes, and adjusted the parameters to make the model suitable for procyclic trypanosomes. Then, for each individual mRNA, we predicted the abundance using the known length and decay rate. For three quarters of procyclic form mRNAs, the predicted and measured abundances differed by less than a factor of 2. The comparison thus enabled us to identify the mRNAs that are less abundant than expected, and are therefore likely to be poorly processed. In addition, we measured the ribosome occupancy of mRNAs under exactly the same conditions as were previously used for the mRNA decay measurements. The combined results enable us to assess the roles of mRNA processing, decay and translation in the developmental regulation of gene expression.

## Methods

### Modelling

The gene expression model [16] was reprogrammed in R-3.1.3 and further optimised as described in the text. All codes are supplied as Additional files 1, 2, 3, 4, 5, 6, 7 and 8. To match experimental data for individual genes with the predictions, we used the mRNA half-lives (in min) and abundances (in number of mRNAs per cell per gene in the haploid genome) that had been measured previously [16], and the most recent estimates of transcript length on TritrypDB (http://tritrypdb.org/tritrypdb/, July 2015). For model optimisation, transcripts with no mapped untranslated regions were excluded. We restricted the data to “reliable” measurements according to the following criteria: (a) The two different estimates of gene copy number for *T. brucei* Lister 427 [16, 25] should differ by less than a factor of 2 and should both exceed 0.5; (b) the error in the half-life estimation (residual mean of squares) must be less than 0.3 [16]; the half-life should be within measurable range (between 5 min and 120 min for bloodstream forms, and 5–240 min for procyclic forms). For bloodstream forms, we had two independent abundance measurements [16, 25]; we stipulated that there should be a less than two-fold difference between them. Three independent measurements were available for procyclic forms; in that case we stipulated that the relative standard deviation should be less than 2. All of these criteria are arbitrary, and could be adjusted. The combined criteria (2-fold each for the gene copy number and abundance measurements) means that we aimed for a maximum error of 4-fold in the number of mRNAs per gene. The final restricted gene lists that met our criteria included 4089 genes for bloodstream forms and 3776 genes for procyclic forms (Additional file 9: Table S1). For bloodstream forms, our attempts to optimise the model were done with more restricted datasets (see Additional file 9: Table S1 and legend to Table 1).

**Table 1:** Parameters used in the different models

		BS-A	BS-B	BS-C	BS-D	BS-E	BS-F	PC-A	PC-B
a	No genes examined	851	851	642	851	851	851	3776	3776
b	μ (min^−1^) Division time	0.0019 (6 h)	0.0019 (6 h)	0.0019 (6 h)	0.0019 (6 h)	0.0019 (6 h)	0.0019 (6 h)	0.0011 (10.5 h)	0.0011 (10.5 h)
c	v (molecules.cell^−1^ .min^−1^)	0.24	0.24	0.24	0.357	0.278	0.278	0.24	0.234
d	k_1_	0.41	data	data	0.41	0.41	data	0.41	0.41
e	k_2_	0.41	0.41	data	0.41	0.41	0.41	0.41	0.41
f	Length adjustment α	z = 600	z = 600	z = 600	z = 660	§	§	z = 600	§
g	Correlation coefficient (r)	0.59	0.60	0.43	0.59	0.54	0.55	0.66	0.60

The Table shows the parameters used for the various models. See also Fig. 1

a) To optimise the model we chose genes for which we had reliable measurements of both half-life and mRNA abundance. For bloodstream forms, we had attempted to use RNASeq data to determine the rate at which the sequences immediately upstream of the splice site disappeared, with the hope of using that as a proxy for the splicing rate [16]. For this form we restricted the dataset to mRNAs that had measured precursor half-times of 1–5 min. For BS-C we narrowed this evern further, using only genes that also had a similar measurement for the gene immediately downstream. This means that the correlation coefficient for BS-C is not directly comparable to those for the other BS models

(b) Based on measured growth rates (see also [17])

(c) The transcription rate (v) is influenced by the initiation and elongation rates; since neither has been measured, the transcription rate was originally calculated for bloodstream forms to obtain the measured steady-state mRNA level for PGKC [17] and this value was used in models BS-A - BS-C. For models BS-D and BS-E the rate was adjusted to give a better fit for the dataset (BS-D) and for BS-E and BS-F, to give a better fit with the new length adjustment (f). For PC-A the rate was left the same as in BS-A. For PC-B we multiplied the adjusted rates from BS-E and BS-F by a factor of 0.84, based on the fact that the DNA replication rate in procyclic forms is 0.84 times that of bloodstream forms [56]

(d) k_1_ = 0.41 is equivalent to a splicing half-time of 1.7 min. This is based on reliable individual measurements of splicing for 3 mRNAs, which gave estimates of 1–3 min [14, 17, 31]. The “data” are the rates estimated by RNASeq [16] (see (a))

(e) Data for BS-C were from the gene immediately downstream (see (a))

(f) For models with a z value: α = (mRNA_length)/z as used in [16]. For models marked §: α = 1/(20.603 x (mRNA_length^-0.461^) for bloodstream forms; α = 1/(343.77 x (mRNA_length^-0.858^) for procyclics. The change in the spliced intermediate over time is:

d([5′ spliced intermediate]/dt) = k_1_ x [precursor] - (k2 + α k_4_ + μ) x [5′ spliced intermediate]

(g) Pearson’s correlation coefficient (r) between the abundances predicted for individual RNAs, and the measured abundances of those mRNAs

The mRNA degradation (k_5_) values for the mRNAs were those measured in [16]. The precursor degradation rates k_3_ and k_4_ were always 0.08 (half-life 8 min) which is the rate of disappearance of the *PGKB* precursor when splicing is inhibited by Sinefungin in bloodstream forms [17]

The transcription rate and mRNA length factor z were both optimised using the Nelder-Mead optimization algorithm, in order to most closely mirror the abundance distribution. As an alternative way to measure the influence of mRNA length on half-life, we re-calculated it as follows: (a) we modelled the expected amount of mRNA without applying any length correction; (b) we divided the measured mRNA abundance by the modelled abundance from (a); (c) we fitted an optimal curve. All of this optimisation was done to fit the overall distribution, rather than for individual genes. Results from the various models are in Additional file 10: Figure S1, Additional file 11: Figure S2 and Additional file 12: Figure S3.

### Motif searches

Motif searches were done using DREME [26], MEME [27] and to analyse pyrimidine patterns, a custom script (Additional file 13). 5′-region comparisons compared 62 mRNAs that had abundances of less than 0.25-fold the predicted value in both forms with 723 mRNAs with abundances of 1.5-fold - 2-fold predicted values. Polypyrimidine tract analysis focused on the 70 nt upstream of the principal splice acceptor, and to reduce the likelihood of considering mis-annotated sites, we considered only mRNAs with annotated 5′-UTRs between 10 and 300 nt. To look for 3′-UTR motifs that affect degradation rates, we tried multiple different categories: for example, mRNAs that are very unstable and stabilised by depletion of XRNA in bloodstream but not procyclic forms, and *vice versa*; mRNAs that are stable in all stages; and various other groupings.

### Trypanosome culture and ribosome profiling analysis

Trypanosomes were cultured exactly as described in [16]. For ribosome profiling, RNA was made using Trizol and trypanosome pellets were snap frozen; both were shipped on dry ice. Ribosome profiling was done exactly as described in [20]. To determine mRNA levels, the mRNA was poly(A) selected, fragmented to pieces of 30–70 nt, then made into cDNA as described previously. It was not possible to conduct a ribosomal RNA depletion as described previously, because the “ribo-minus” kit that had been used is no longer available and the new version is unsuitable for trypanosome RNA. Comparative analyses concentrated on a set of genes that represented each unique open reading frame [9, 16].

To analyse polysomal RNA, trypanosome extracts were subjected to sucrose density gradient centrifugation [28]. RNA was prepared from the fractions that were denser than monosomes; the monosomes; and lighter fractions. A portion was used for Northern blotting with the spliced leader as probe, to quantify the proportion of mRNA in the polysomes. Ribosomal RNA was depleted from another aliquot by incubation with oligonucleotides complementary to trypanosome rRNA together with with RNase H, and the RNA was sequenced as described previously. Further details of this polysomal RNA analyses will be published elsewhere.

Alignments were done using custom scripts, as previously described [28, 29]. Statistical analyses were done in R or RStudio. To find differences between datasets we used DeSeq [30]. Plots were made using R and Microsoft Excel, then re-formatted in Adobe Illustrator.

## Results

### Adjustments to the gene expression model and adaptation to procyclic forms

In our previous publication, our model for bloodstream-form gene expression was used to simulate transcriptome-wide mRNA abundances, using half-life and mRNA length distributions that matched the measured values [16]. We did not, however, attempt to analyse predictions for individual mRNAs. Before doing that, we decided to examine various details of the model in order to make sure that it reflected the data to the best extent possible. Since we have data for mRNA half-lives, the parameters that needed to be optimised were the transcription rate, splicing kinetics, polyadenylation kinetics and the relationship between mRNA length and precursor decay. To optimise the model we compared its output with data for mRNAs for which we have reliable measurements of gene copy number, mRNA abundance, mRNA length, and mRNA half-life. After each model adjustment, we first compared the predicted population distribution with the real population distribution. Results are shown in the left-hand panels of Additional file 10: Figure S1, Additional file 11: Figure S2 and Additional file 12: Figure S3. Next, for each individual mRNA, we compared the simulated mRNA abundance with the measured abundance: these results are in the right-hand panels of Additional file 10: Figure S1, Additional file 11: Figure S2 and Additional file 12: Figure S3. Table 1 shows the changes made in the different model versions, with Pearson correlation coefficients at the level of individual mRNAs.

In the next step, we looked at processing in bloodstream forms. Reliable splicing kinetic measurements are available for just 3 genes: a half time of 1.7 min for the *PGK* precursor in bloodstream forms [17], and similar values for *HSP70* in bloodstream forms [31] and for alpha and beta tubulin in procyclic forms [14]. We first used the published bloodstream-form model [16] with no modifications (Table 1, column BS-A). We inputted the half-life and mRNA length distributions from 851 genes (for the choice see below), and set splicing and polyadenylation half-times to 1.7 min. The Pearson correlation coefficient between predicted mRNA abundance and real abundance, at the level of individual mRNAs, was 0.59 (Table 1).

In our previous RNASeq analyses, we had attempted to measure bloodstream-form splicing half-times transcriptome-wide, using as a surrogate the rate at which the precursor disappeared [16]. We did not know whether these measurements had any validity because the numbers of RNASeq reads from precursors were low, and many mRNAs have several splice acceptor sites. Nevertheless, for model optimisation, we focused on 851 genes for which we had estimated splicing half-times of between 1 and 5 min. We hoped that in this way, we would be excluding mRNAs with very slow processing, since this might interfere with model optimisation. Instead of using a constant splicing time, we tried inserting the values for processing kinetics that had been measured by RNASeq (Table 1, columns BS-B and BS-C; Additional file 10: Figure S1). The correlation was not improved relative to BS-A, which confirmed our previous suspicions that our attempted transcriptome-wide measurements of splicing rates for individual genes were too inaccurate to use in modelling. We therefore decided, for subsequent models, to use a fixed splicing time of 1.7 min. We started from model BS-A and optimised the transcription rate (v) and the length correction (z-factor) simultaneously using the Nelder-Mead optimization function (Table 1, column BS-D; Additional file 10: Figure S1). This yielded a similar fit to model BS-A, both for the overall distribution and for individual mRNAs.

The last parameter to be considered was the relation between length and abundance. We needed to assess whether this relationship was more complex than the simple hyperbolic relation used so far. To obtain an empirical answer, we plotted abundance against half-life for the 851 genes and fitted the length correction directly. The power relation that we obtained (see formula in Fig. 1 and notes to Table 1) fitted the data *globally* better than the linear relation used before, but slightly decreased the correlation between prediction and measurement at the level of *individual* mRNAs (Table 1, columns BS-E and F; Additional file 11: Figure S2). This adjustment also changed the calculated transcription rate.

Next, we made two different models for procyclics. One, PC-A, was based on BS-A [16], and for the other (PC-B) we fitted a length-abundance power relation as for BS-E and F. We used a longer cell division time, as procyclic forms grow slower than bloodstream forms. We calculated the steady-state mRNA levels for 3776 genes with both models. Comparisons between simulated and real abundances for both models are in Additional file 12: Figure S3. Both models fitted the procyclic data considerably better than any of the bloodstream-form models did for the bloodstream-form data (Table 1, Columns PC-A and PC-B).

### Comparing simulated and real abundances of individual mRNAs reveals which transcripts are regulated by processes other than mRNA decay

**Fig. 2:**
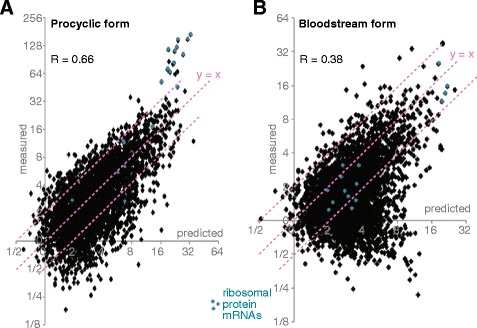
Correlation between predicted and measured mRNA amounts. The numbers of mRNAs per cell were predicted for the subset of mRNAs with reliable measurements of gene copy number, half-life, and abundance. The predictions were then compared with measured values of mRNA abundance for (**a**) procyclic forms (model PC-A) and (**b**) bloodstream forms (model BS-D). Each diamond represents a different open reading frame. Correlation coefficients are for log_2_-transformed values. The pink dotted lines are for y = 2x, y = x and 2y = x. Results for ribosomal protein genes are in cyan

We used some of the better-performing models to predict the abundances of all mRNAs for which reliable half-life and mRNA length data were available. Comparisons for individual mRNAs using models PC-A and BS-D are in Fig. 2; other results are in Additional file 14: Table S2 and Additional file 11: Figure S2 and Additional file 12: Figure S3. The fit was strikingly better for procyclic forms than for bloodstream forms. The good fit for procyclic forms indicates that the structure of our model is appropriate. Using model PC-A, 74 % of all mRNAs had abundances that differed from the predictions by less than 2-fold, and 96 % differed by less than 4-fold. Where discrepancies were present, the mRNAs were usually less abundant than expected. Intriguingly, of the 15 mRNAs (0.4 %) that were more than 4-fold more abundant than predicted in procyclic forms, 6 encoded ribosomal proteins, and an additional 6 ribosomal protein mRNAs were more than 3-fold more abundant than predicted. The 20 mRNAs encoding ribosomal proteins in the dataset are indicated in cyan in Fig. 2a; the other mRNAs in this category were too stable to allow half-life measurement [16]. The observed:predicted ratios of this functional class in procyclic forms were significantly higher than all of the other classes (*p* = 0.008 by ANOVA with Holm-Bonferroni correction). We confirmed that the automatically-calculated half-lives for the 6 most clearly over-represented mRNAs were not erroneously short: this was not the case since the maximal discrepancy was a calculated half life that was about 20 % shorter than a manually plotted one (Additional file 15: Figure S4). No other class showed such a strong bias (Additional file 16: Figure S5). Since these RNAs are all relatively short we looked to see whether the same effect was seen using the power relation length adjustment (model PC-B); in this case, 32 mRNAs were more abundant than predicted and 13 of them encoded ribosomal proteins (Additional file 12: Figure S3, Additional file 9: Table S1, sheet 2). This result suggests that the mRNAs encoding ribosomal proteins are either very rapidly processed, or abnormally resistant to precursor degradation.

Model BS-D was used to predict the abundances of the 4088 individual mRNAs with reliable measurements in bloodstream forms. As we previously noted [16], many mRNAs were less abundant than expected (Fig. 2b). Just 3 mRNAs were more than 4 times more abundant than was predicted by the model, and this time the ribosomal protein mRNAs did not show unusual behaviour. 452 mRNAs were more than 4 times less abundant than predicted for bloodstream forms, as opposed to 127 in procyclic forms. A lower than expected abundance indicates that one or more of the parameters that was used in the modelling for that gene is incorrect. The most likely explanation is that the processing half-time is longer than 1.7 min, since processing can be modulated at the level of individual genes. However, slower transcription or an enhanced susceptibility to nuclear degradation also must be considered.

**Fig. 3:**
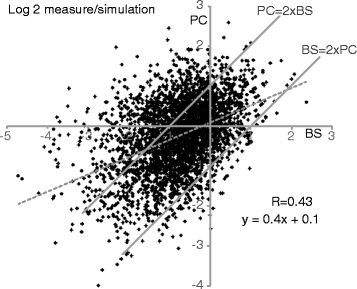
Relationship between prediction and measurement for bloodstream and procyclic forms. For each unique gene considered, the amount of mRNA that was measured was divided by the predicted amount, and the log_2_ of the values for both stages plotted. BS - bloodstream form, model BS-D; PC - procyclic form, model PC-A. A value of 0 indicates that the prediction was perfect; this could however also be true if transcription was faster, and processing slower, than the values that were set in the model. Measures above 0 indicate either that a processing half-time is shorter than 1.7 min, or that the steady-state transcription rate is greater than the value that was used for the model. values below 0 indicate that the splicing or polyadenylation half-time is longer than 1.7 min. Spots above the line “PC = 2xBS” are mRNAs for which the measured/prediction results was at least 2-fold higher in procyclic than in bloodstream forms; those below the “BS = 2xPC” line have better processing in bloodstream forms than in procyclics

To find out which genes showed low abundance only in one of the life-cycle stages, we selected the 3381 genes for which reliable measurements for both stages were available. For each mRNA, we then divided the measured mRNA amount by the predicted amount. Figure 3 shows a comparison of the ratios for bloodstream and procyclic forms. For 68 % of the mRNAs, the ratios differed less than 2-fold, and 94 % differed less than 4-fold. 50 mRNAs were more than 4 times less abundant than predicted in both forms, 63 were less abundant only in procyclics, and 265 in bloodstream forms (Additional file 14: Table S2).

We compared the RNAs that were less abundant than expected with the remainder of the mRNAs. No functional category was significantly enriched, and genes at the end of transcription units (defined as last three genes before a transcription stop region) were not over-represented. If processing were slower than normal, either splicing or polyadenylation could be affected. Polyadenylation sites are too heterogeneous for automated analysis, but we could analyse the sequences upstream of the open reading frames and dominant splice acceptor sites. There were no significant differences between the groups in the lengths of the polypyrimidine tracts upstream of the splice sites, and no enriched 5′-UTR motifs. In contrast, mRNAs that showed unexpectedly low abundance either in both forms, or in procyclics only, had significantly longer annotated 5′-UTRs than those with “normal” abundance. The transcriptome-wide median 5′-UTR length was estimated at 130 nt [10]. We found a median length of 101 nt for mRNAs that were not under-represented in either stage; 181 nt for mRNAs that were of low abundance in both forms (Student t-test probability that the distribution is identical to the first set was *p* = 0.001) ; and 144 nt (*p* = .02) for those with low abundance in procyclics only. There was no significant enrichment of upstream open reading frames in any of the categories. Notably, the median 5′-UTR length for ribosomal protein mRNAs is 22 nt.

### Modelling does not yield evidence for regulation of polymerase II transcription

There are three sets of experimental evidence that RNA polymerase II transcribes Kinetoplastid genomes at a constant rate, independent of the chromosome, transcription unit or individual gene. The most convincing results are from nuclear run-on experiments done with *Leishmania*, but quantitation of such experiments is difficult because the signals are rather low [3, 4]. Second, in *T. brucei* procyclic forms, integration of a reporter plasmid into four different positions in polymerase II transcription units, upstream of genes encoding tubulin, aldolase, actin, and HSP70, yielded similar levels of expression [32]. Thirdly, bloodstream-form mRNA abundance:half-life ratios across chromosome 10 do not show obvious differences between transcription units [16].

We decided to use our model to challenge the hypothesis of equal transcription rates quantitatively. We used data from procyclic forms because overall, the model fits the data better in this form (Table 1 & Fig. 2b). Using published histone modification results to find start and stop sites [5] (TritrypDB.org), we selected transcription units from chromosomes 9, 10 and 11 that contained at least 13 genes with “reliable” half-life and abundance measurements. Starting from model PC-B, we allowed the transcription rate to vary in order to get an optimal fit of the results to the data. Interestingly, the resulting calculated transcription rates varied over nearly an order of magnitude (Table 2, Additional file 17: Table S3). This could be due to genuine differences in transcription rates, or could be a consequence of variations in processing across the units.

**Table 2:** Optimised transcription rates for selected polycistronic units. Rates of transcription were optimised using mRNA half-life and abundance data to get the best fit, using model PC-B. For each pair of transcription units, divergent transcription initiates within a common region

Polycistronic unit pair	Unit	Optimised Transcription rate	Pearson correlations (R)	Number of genes
Tb927_10_p1	1	0.51	0.51	28
	2	0.20	0.55	36
Tb927_10_p2	1	0.26	0.73	31
	2	0.15	0.66	36
Tb927_10_p3	1	0.26	0.66	30
	2	0.15	0.67	34
Tb927_10_p4	1	0.28	0.73	36
	2	0.07	0.36	52
Tb927_10_p5	1	0.24	0.66	40
	2	0.13	0.67	26
Tb927_11_p1	1	0.05	0.73	27
	2	0.20	0.65	41
Tb927_11_p2	1	0.12	0.66	36
	2	0.45	0.3	18
Tb927_9_p1	1	0.14	0.81	35
	2	0.08	0.59	56
Tb927_9_p2	1	0.43	0.59	37
	2	0.03	0.74	13
Tb927_9_p3	1	0.61	0.36	18
	2	0.34	0.71	14

Next, we examined paired divergent transcription units that share a common initiation region [10]. If transcription really varies, and the initiation rate is determined only by histone modifications around the start sites, then units that share a promoter region should show similar predicted transcription rates. Contrary to this expectation, the transcription rates that were fitted for units sharing an initiation region did not correlate. There are two possible interpretations of this result. One possibility is that in divergent transcription units, the rates of transcription in the two directions are not equal. The alternative is that by chance, different transcription units have different average processing rates. Only direct measurements of transcription can resolve this issue.

### Ribosome loading in procyclic forms is affected by the growth conditions

The original aim of our project was to allow transcriptome-wide modelling of gene expression from RNA synthesis to the final steady-state protein level. For this, we needed to measure translation and protein degradation. Two previous analyses of translation by ribosome profiling have revealed very wide differences in mRNA translation efficiencies [20, 21]. To compare models with quantitative data, however, it is essential that the cellular growth conditions for all measurements should be identical. To extend our analysis to translation, we therefore cultured trypanosomes exactly as we had for the measurements of mRNA decay [16] and subjected samples to ribosome profiling [20]. We analysed three procyclic-form and two bloodstream-form samples (Additional file 18: Table S4). Correlations between similar samples were always greater than 0.96 (not shown).

**Fig. 4:**
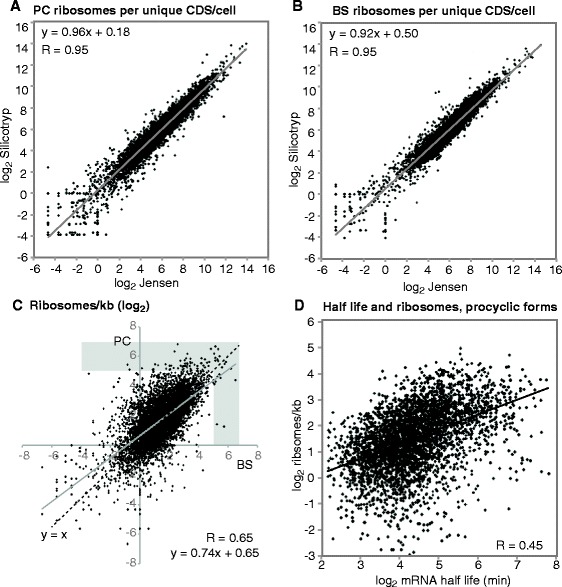
Ribosome profiling results. **a** Ribosomes per coding sequence (CDS) per cell, procyclic forms (PC). Our previously published results (labelled “Jensen”) [20] are compared with the set reported here (labelled “Silicotryp”). **b** As in (**a**) but for bloodstream forms (BS). **c** Developmental regulation: the numbers of ribosomes per kilobase are compared from bloodstream and procyclic forms. The grey-shaded area indicates impossible densities of less than 30 nt per ribosome. **d** The relationship between mRNA half-life and ribosome density for procyclic forms. Only mRNAs with reliable measurements of abundance and half-life were considered, and impossible ribosome densities were excluded

We first compared the new “Silicotryp” results from the ribosome profiling with our previous ones (denoted “Jensen” in Figures and Tables). Overall, the results correlated well (Fig. 4a, b). After excluding VSG expression site genes, only 22 open reading frames showed at least two-fold, statistically significant differences for bloodstream forms (Additional file 18: Table S4, sheet 4). This is not surprising since the trypanosome strain and culture conditions were almost identical. In contrast, for the procyclic forms 314 genes showed significant differences (Additional file 18: Table S4, sheets 5 and 7).

The total numbers of ribosomes per cell that mapped to each ORF is a measure that combines mRNA level and ribosome density. Interpretation of the results from procyclic forms is complicated because there were differences in several parameters: trypanosome strain, medium composition, and cell density. The Jensen results are from strain TREU927, grown to densities of 5–10 × 10^6^/ml in SDM79 medium, whereas the Silicotryp [19] results are for strain Lister 427, grown to densities of 1–2 × 10^6^/ml in MEM Pros. Metabolites from 10 % serum are present in both media. MEM Pros has 5 mM proline as an energy source, with about 500 μM glucose from the foetal calf serum, whereas SDM79 also contains 10 mM glucose. MEM Pros contains only adenosine as a purine source, whereas SDM79 also has guanosine and small amounts of pyrimidines.

Procyclic forms grown with abundant glucose are known to convert it mainly to succinate and acetate, with limited dependence on respiratory complexes II and IV and the F1 ATPase [33]. Proline catabolism results mainly in secretion of alanine and glutamate, but succinate is also subject to complex-II-dependent channelling towards pyruvate and gluconeogenesis [33]. The differences that we saw in the two datasets ran contrary to our expectations. We expected the proline-grown cells to express more proline dehydrogenase [34], since the mRNA level is higher [16] but no significant differences were observed in the ribosome-bound mRNAs for this or any other enzyme of proline catabolism. Strangely, the proline-dependent MEM-Pros-grown cells showed *lower* translation of several components involved in mitochondrial electron transport, but *greater* translation of three proteins involved in glucose metabolism - hexokinase and two subunits of the mitochondrial alternative oxidase.

The increased production of several other proteins in the MEM-Pros grown parasites, such as those involved in kDNA replication, the kinetochore, and DNA repair could be linked to lower cell density, rather than medium composition. This population also had increased translation of adenosine and guanosine kinases, and of an uncharacterised procyclic-form-specific purine transporter. Differences in expression of some RNA-binding proteins might have been responsible for some of the differences in mRNA abundance or translation. For example, excess translation of the stress response regulator ZC3H11 was seen in the MEM-Pros grown parasites, compatible with the observed higher production of HSP83 and mitochondrial HSP60 [35].

### Ribosome density does not correlate with mRNA half-life

**Fig. 5:**
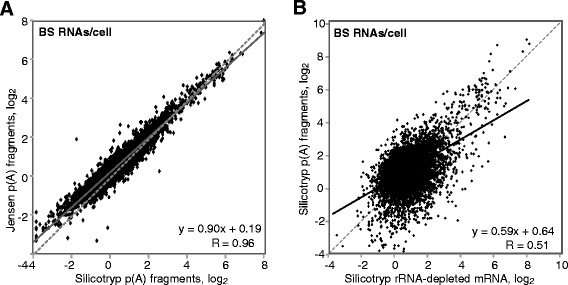
Effect of methods on the measurement of mRNA abundance. **a** Poly(A) + mRNA from bloodstream forms, fragmented to 30–70 nt before sequencing; comparison of Silicotryp results vs Jensen results [20]. **b** Poly(A) + RNA from bloodstream forms, 30–70 nt fragments compared with published results for rRNA-depleted (ribo-minus) RNA [16], prepared from bloodstream forms cultured under identical conditions

To calculate ribosome densities, the number of ribosomes has to be divided by the mRNA abundance. We had two alternative datasets for this: the published results for rRNA-depeted mRNA (sequenced as 300 nt fragments), and, for bloodstream forms, results from more highly fragmented (30–70 nt) poly(A) selected mRNA. (Similar results for 30–70 nt fragments from procyclic forms showed 3′ bias so were discarded.) The RNA used for ribosome profiling was from 30 to 70 nt fragments but was not poly(A) selected. It is well known that the methods used to remove rRNA, and to build libraries, can substantially influence RNASeq results [36–38] and we too found considerable differences between rRNA-depleted and poly(A) selected trypanosome transcriptomes [25]. As expected, the new “Silicotryp” reads for highly fragmented bloodstream form poly(A) + mRNA correlated quite well with those generated previously by the same method [20] (Fig. 5a), but less so with those from rRNA-depleted mRNA (Fig. 5b). Since we already had half-life results for rRNA-depleted mRNA, we decided to use those abundances for subsequent calculations.

Ribosome densities were calculated for bloodstream forms (Additional file 19: Table S5, sheet 2 column O) and procyclic forms (Additional file 19: Table S5, sheet 1 column K). For bloodstream forms, only 22 mRNAs had impossible ribosome densities (less than 30 nt/ribosome); for procyclics there were 69 (Fig. 4c). Such results could be caused by an under-estimation of the number of mRNAs; read counts can also be inaccurate if a gene is internally repetitive or part of a multi-gene family. The “impossible” densities could also be caused by the fact that we assumed that all ribosomes are engaged in translation, whereas in fact, a small portion is present as subunits and we do not know how many monosomes are bound to mRNA (see Legend to Additional file 19: Table S5). The ribosome densities given in this paper are therefore almost certainly over-estimated. The median ribosome density that we calculated was 3.2/kb for procyclics and 2.7/kb for bloodstream forms. Most mRNAs showed similar translation in both stages; 1615 mRNAs were at least 2-fold better translated in procyclic forms, and 629 in bloodstream forms (Fig. 4c). As previously noted [20, 21], there was almost no correlation between mRNA level and ribosome density (not shown).

When mRNAs were split into broad functional categories, there was no significant developmental regulation of ribosome density for any category. We also found few significant differences in ribosome densities between the categories (Additional file 20: Figure S6). In bloodstream forms, mRNAs encoding proteins of glucose and glycerol metabolism had significantly higher ribosome densities than average, with a median of about 130 nt/ribosome. The subset involved in glycolysis were closely packed, with only 50 nt/ribosome, and was also well-translated in procyclics (73 nt/ribosome). Results for alpha and beta tubulin and actin were similar. Citric acid cycle and pentose phosphate pathway mRNAs were also well translated in both forms. Since the citric acid cycle enzymes show no activity in bloodstream forms, it is likely that some of these mitochondrially-targeted proteins are degraded after translation, as was observed for cytochrome c [39]. As previously observed [20], most mRNAs encoding ribosomal proteins are not particularly well translated (2.8 ribosomes/kb in procyclics, 1.5 in bloodstream forms) although they are abundant and very stable.

The availability of ribosome density and mRNA half-life measurements for cells grown under identical conditions meant that we could now ask questions about how the two processes interact. We therefore examined the hypothesis that packing of ribosomes on the mRNA is correlated with the mRNA half-life. The correlation between ribosome density and half-life in procyclic forms was extremely weak (Fig. 4d, Additional file 19: Table S5, sheet 3), and there was none for bloodstream forms (Additional file 19: Table S5, sheet 4). We therefore decided to look at the mRNAs with half-lives that had been too long to measure using transcription inhibition and RNASeq. Interestingly, the average density on these coding regions was 11–13 ribosomes/kb, whereas the average densities on the mRNAs with measureable half-lives were 5.2 ribosomes/kb for procyclics and 3.7 ribosomes/kb in bloodstream forms. This would be compatible with the idea that dense ribosome packing stabilises an mRNA, but could also simply indicate that these mRNAs have been selected for both high stability and very efficient translation.

Some effects on ribosome loading are passive. A very unstable mRNA cannot have a high ribosome density because it takes time for translation to initiate after an mRNA exits the nucleus [40]. If the mRNA is decapped first, no initiation will occur. In yeast, the interval between initiation events varies from 4–233 s, with a median of 40 s [41]; on average, an mRNA with a half-time of 5 min and a 40s initiation interval cannot load more than 7 ribosomes before it is decapped. Those mRNAs with long open reading frames and short half-lives would thus be expected to show particularly low ribosome densities. There was, however, no correlation between coding region length and ribosome density, either for the entire dataset or for mRNAs with half-lives of 5–6 min. This suggests that sequence-specific regulatory mechanisms override passive kinetic effects.

### In procyclic forms, low average ribosome densities are partially due to lack of translation initiation

**Fig. 6:**
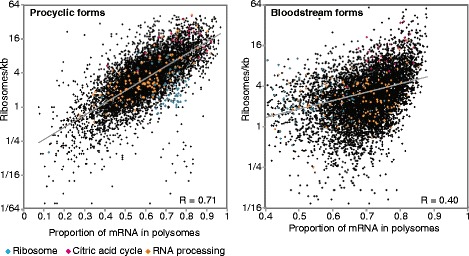
Ribosome density measurements reflect variable loading of mRNA on polysomes. The number of ribosomes per kb of total mRNA (note log scale) is plotted against the proportion of the same mRNA that is in the polysomal fraction. Results for specific functional classes are highlighted in different colours

Ribosome profiling measures the average ribosome occupancy across the mRNA population. A low ribosome density could mean that a small proportion of mRNAs is highly translated and the rest are stored; or it could mean that every mRNA has a low number of ribosomes. If only half of the mRNAs are translated, but elongation is slow or ribosomes are stalled, the overall calculation might give a high average ribosome density. To distinguish these possibilities we fractionated polysomes on sucrose gradients, and measured the levels of each mRNA in the polysomal, monosomal and free fractions by RNASeq. The proportion of total mRNA in the fractions was also measured by Northern blotting and hybridisation with a spliced leader probe. This measurement was used to normalise the RNASeq data, and the proportion of each mRNA in the polysomal fraction was calculated. For procyclic forms, we found a partial correlation between the proportion of mRNA on polysomes and the ribosome density (Fig. 6). This suggests that some of the translation regulation is at the level of initiation. We examined this in more detail for mRNAs encoding some functional protein classes. For proteins with functions in mRNA processing (Fig. 6, orange dots), there were on average 5 ribosomes/kb in the total mRNA, but if we re-calculated this including only the fraction of mRNA that is on polysomes, there were 7 ribosomes/kb. Citric acid cycle mRNAs were mainly in polysomes and had higher ribosome densities: on average 22 ribosomes/kb in total, and 29 ribosomes/kb in polysomal RNA (Fig. 6, pink dots). mRNAs encoding ribosomal proteins, in contrast, were mostly on polysomes (Fig. 6a, blue dots) but with an average of only 4 ribosomes/kb in polysomal RNA. In contrast, results from bloodstream forms (Fig. 6b) revealed no correlation between the percentage of the mRNA on polysomes and the ribosome density measured by RNA-Seq. This could mean that bloodstream trypanosome mRNAs show more extensive variations in translation elongation rates than do procyclic mRNAs.

## Discussion

The long-term aim of our project is to explain quantitatively the process of trypanosome gene expression from DNA to protein at steady state. Trypanosomes are a good model for this because of their polycistronic transcription - which means that we can, at least initially, assume that transcription is constant for all genes at steady state. The number of steps is also limited. As with all mathematical models of biological processes, some steps can be fed with detailed measurements, whereas for others, parameters have to be modelled to fit the existing data as well as possible. In the case of the phosphoglycerate kinase locus, nearly all parameters could be measured: cell division time, splicing kinetics, mRNA and protein levels and half-lives, and ribosome loading. This allowed the construction of a kinetic model that could be used to estimate the transcription rate and the polypeptide elongation rate [17]. This can readily be extended to other individual genes for which measurements are available [16]. Our ability to extend the model to the entire proteome has so far been constrained by technical limitations: we lack proteome-wide measurements of protein abundance and turnover. Consequently, our genome-wide modeling can currently cover only the steps from the genome to steady-state mRNA levels. Since the best-measured kinetic parameters are the cell division time and the mRNA half-lives, we are able to adjust the model to simulate mRNA abundances, and then use it to predict variations in the other parameters.

In this work, we showed that most of the variation in mRNA abundances in procyclic forms can be predicted based on differences in mRNA half-life and in length-dependent precursor degradation. Only 6 % of mRNAs had abundances in procyclic forms that differed more than 4-fold from the predicted value, and most of those were less abundant than expected. This must be because the mRNA spends longer time as a precursor than is assumed in the model, resulting in excess precursor degradation. This could, in turn, happen because transcription elongation, *trans* splicing, or polyadenylation are slower than the rates that we fed into the model. Slower processing seems the more likely explanation. The processing half-time that we used in the model was derived from rates that were measured for three very abundant mRNAs. There is plenty of evidence that splicing efficiencies can be influenced by the sequences around the splice acceptor site [42–44]. We found that mRNAs with unexpectedly low abundance in procyclics had somewhat longer annotated 5′-UTRs than normal. The presence of an open reading frame downstream of the splice site is important in splice site choice [43, 44], so it may be that splice acceptor sites that are further from the start codon are used less efficiently.

Deviations from the model predictions were much more marked for bloodstream-form trypanosomes than for procyclics, implying that developmental regulation of processing could be biased towards reduced abundance of mRNAs that are not required in bloodstream forms. Such notions need to be tested by more accurate measurements of processing kinetics. These measurements are technically demanding, but our model predicts which mRNAs are good candidates for such experiments. For example, there are six mitochondrial protein mRNAs that have similar half-lives in bloodstream and procyclic forms, but are much less abundant in bloodstream forms (Additional file 14: Table S2, sheet 2). There have been several recent reports of trypanosome factors that can influence *trans* splicing [45–48] and it will be interesting to find out whether these target such mRNAs.

Our ribosome profiling experiments confirmed the major role of translation control in trypanosomes, with huge variations between mRNAs in ribosome densities. Unfortunately these results alone cannot be used to predict the translation rate [41], since a high ribosome density could indicate not only high translation, but also pausing or slow elongation. However, in *E. coli* and yeast, ribosome profiling data correlates well with protein abundance both within and across coding regions, suggesting that in many cases ribosome profiling approximates absolute protein production [49]. In procyclics, there was a weak correlation between the ribosome occupancy of mRNAs and the proportion of the mRNA that co-migrated with polysomes. This suggests that at least part of the variation in ribosome density is caused by regulated translation initiation: some mRNAs are being translated, whereas others are not. Some of the variation could be between cells at different cell-cycle stages. To understand this further it would be interesting to subject synchronised cell populations, and specific portions of polysome gradients, to ribosome profiling.

We observed a moderate correlation between mRNA half-life and ribosome density in procyclic trypanosomes. There is extensive evidence for cross-talk between translation and the mRNA decay machinery in yeast; although polysomal mRNAs are substrates for the decay pathway [50, 51], there are several circumstances under which mRNAs with poor translation show accelerated decay [52]. However a correlation between ribosome density and mRNA half-life does not necessarily mean that high ribosome densities *cause* an mRNA to be stable. It may just be that mRNAs that encode abundant proteins are selected for both high abundance and efficient translation.

The regulation of ribosomal protein synthesis seems to be exceptional in at least two ways, especially in procyclic forms. Most of these mRNAs are too stable to allow a half-life measurement in our RNASeq analysis. Those with measurable half-lives, however, were more abundant than expected in procyclic forms, suggesting either very rapid processing or unusual resistance to co-transcriptional degradation. The second peculiarity, which has been noted before [20], is that the ribosomal mRNAs have strikingly low ribosome densities, although high proportions of these mRNAs are in polysomes. Why could growing cells need extremely efficient production of a stable mRNA, then not use it? Most ribosomal protein mRNA levels are highest in early G1 [53]; perhaps their translation is also cell-cycle regulated. Alternatively, or in addition, the surplus mRNA may enable rapid synthesis of extra ribosomes when trypanosomes move to a new environment. Interestingly, the “exceptionality” of ribosomal protein mRNAs is not confined to trypanosomes. Ribosomal protein mRNAs also have low ribosome densities in yeast [54], just as in trypanosomes, but they also show an interesting peculiarity in mRNA synthesis and decay. Miller et al. studied measured transcription and mRNA decay rates in yeast, by in vivo 4-thiouracil labelling followed by RNASeq [23]. They found that in general, mRNA synthesis and decay rates were not correlated for individual mRNAs. In contrast, ribosomal protein genes showed exceptionally high transcription rates, with a strong correlation between their rates of mRNA synthesis and degradation.

The comparison between our modelling results and the data indicate that whereas procyclic-form trypanosomes rely mainly on mRNA decay to determine mRNA levels, many mRNAs in bloodstream-form trypanosomes are subject to an additional layer of regulation. Similarly, whereas the results for procyclic forms suggest that translation is often controlled at the level of initiation, bloodstream-form translation control is more complex. It is generally thought that mammalian-infective trypanosomes evolved from an ancestor that infected only arthropods [55]. Our results suggest that adaptation to the mammalian environment may have involved the acquisition of novel gene expression control mechanisms that operate in the nucleus.

### Ethics

This study did not include the use of any animals, human or otherwise, so did not require ethical approval.

## Conclusions

Levels of mRNAs in procyclic form trypanosomes are determined mainly by length and mRNA decay, with some control of precursor processing. In bloodstream forms variations in nuclear events play a larger role in transcriptome regulation, suggesting aquisition of new control mechanisms during adaptation to mammalian parasitism.

### Availability of supporting data

The polysome gradient data are available under accession numbers E-MTAB-4558 (bloodstream forms) and E-MTAB-4555 (procyclic forms). The ribosome profiling results are at NCBI with bioproject ID PRJNA315042.

## Additional files

Additional file 1: Script for model BS-A (R 2 kb) Additional file 2: Script for model BS-B (R 2 kb) Additional file 3: Script for model BS-C (R 2 kb) Additional file 4: Script for model BS-D (R 2 kb) Additional file 5: Script for model BS-E (R 2 kb) Additional file 6: Script for model BS-F (R 2 kb) Additional file 7: Script for model PC-A (R 2 kb) Additional file 8: Script for model PC-B (R 2 kb) Additional file 9: Table S1. Datasets used for modelling, with results. Data are taken from [16]. Sheet 1: We chose coding regions for which there had been no more than 2-fold differences in either copy number, or mRNA abundances, between 2 different experiments in bloodstream forms. The error in the half-life measurement in bloodstream forms (residual sum of squares) was also less than 0.3. Sim = simulated results; results for model BS-D (columns L & M) were used for the main analyses but those for BS-A and BS-E are also included in columns R-U. Column P has the following categories: “1. High”: mRNA amount is at least 4 times higher than expected; “2. Low”: mRNA amount is at least 4 times less than expected; 3. Normal: mRNA amount is less than 4 times more or less than expected. Column Q is like column P except that the boundary is within 2-fold. Sheet 2: As for sheet 1, but for procyclic forms. In this case mRNAs were taken for which the standard deviation of 3 abundance measurements was less than twice the mean. Sim = simulated results; results for model parameters PC-A (columns L & M) were used for the main analyses but those for PC-B are also included in columns R&S. Sheet 3: Coding regions from sheet 1 for which there was a measured bloodstream-form splicing time of less than 5 min; used for testing all bloodstream-form models except BS-C. Sheet 4: Coding regions from Sheet 4 for which the next gene downstream also had a measured splicing time of less than 5 min; used for testing bloodstream-form model BS-C. (XLSX 1679 kb) Additional file 10: Figure S1. Adjustments to the gene expression model for bloodstream forms. The graphs on the left show mRNA abundances plotted against their half-lives. Modelled mRNA abundances are in red, and the real mRNA abundances are in black. The green bar shows the 95 % confidence interval and blue shadowed area shows the 95 % prediction band. In the graphs on the right, the real mRNA abundances are plotted against the simulated values for the same half-life and mRNA length. The values and models used for the predictions are listed in Table 1. Log_2_ values were used. (PDF 1371 kb) Additional file 11: Figure S2. Further adjustments to the gene expression model for bloodstream forms. Top four panels: The graphs on the left show mRNA abundances plotted against their half-lives. Modelled mRNA abundances are in red, and the real mRNA abundances are in black. The green bar shows the 95 % confidence interval and blue shadowed area shows the 95 % prediction band. In the graphs on the right, the real mRNA abundances are plotted against the simulated values for the same half-life and mRNA length. The values and models used for the predictions are listed in Table 1. Lower panels: These are the same as Fig. 2B, except that models BS-A and BS-E were used and log_2_ values are shown. (PDF 1142 kb) Additional file 12: Figure S3. Adjustments to the gene expression model for procyclic forms. Upper four panels: The graphs on the left show mRNA abundances plotted against their half-lives. Modelled mRNA abundances are in red, and the real mRNA abundances are in black. The green bar shows the 95 % confidence interval and blue shadowed area shows the 95 % prediction band. In the graphs on the right, the real mRNA abundances are plotted against the simulated values for the same half-life and mRNA length. The values and models used for the predictions are listed in Table 1. Lower panel: This is like Fig. 2a, showing the ribosomal protein mRNAs, except that the model used is PC-B and log_2_ values are shown (PDF 1630 kb) Additional file 13: Script for polypyrimidine tract analysis (R 2 kb) Additional file 14: Table S2. Sheet 1: Results for coding regions present in both sheets 1 and 2 of Additional file 9: Table S1 are compared, using PC-A and BS-D for modelling. Column “BS ratio/PC ratio” is column M divided by column H and indicates possible developmental regulation of processing. Column O: “1. BS low”: mRNA amount is at least 4 times less than expected in bloodstream forms; “2. PC Low”: mRNA amount is at least 4 times less than expected in procyclic forms; 3. Both low: mRNA amount is at least 4 times less than expected in both forms; 4. Normal: remaining genes, except for “5. BS high”: mRNA amount is at least 4 times more than expected in bloodstream forms. Data for 5′-UTRs and upstream ORFs are from TritrypDB. Sheet 2: mRNAs that were at least 4 times less abundant than predicted in bloodstream forms, and more abundant than predicted procyclic forms. Sheet 3: mRNAs that were at least 4 times less abundant than predicted in both bloodstream and procyclic forms. Note that columns Q and R are not independent since each includes the mRNA abundance. Sheet 4: Statistical analyses. Sequence information was taken from from TritrypDB. (XLSX 796 kb) Additional file 15: Figure S4. Accuracy of automatically calculated half-lives and abundances for mRNAs encoding ribosomal proteins. The original mRNA decay results for six ribosomal protein mRNAs that were more than 4 times more abundant than expected in procyclic forms are shown, as reads per million reads corrected for total mRNA abundance [16]. The key is on the Figure. The red arrow indicates the half-life that was calculated in [16] according to [57]. (PDF 423 kb) Additional file 16: Figure S5. Ratio between observed and predicted mRNA abundances in procyclic forms, for mRNAs encoding proteins in different functional categories. The categories were manually assigned and can be found in the supplementary tables. The box plot indicates the median with first and third quartile, with whiskers for the 95 % confidence limits. The ANOVA p-value for ribosomal protein mRNAs was 0.001. (PDF 395 kb) Additional file 17: Table S3. Transcription rate analysis. Transcription rates were fitted for genes in individual polycistronic transcription units that share a common transcription initiation region. The lists of genes used are in the following sheets. The top sheet also shows calculations for some other transcription units. (XLSX 43 kb) Additional file 18: Table S4. Ribosome profiling data. Jensen - data from [20]; Silicotryp - data from [16] and this paper. All numbers were suitably rounded after the calculations had been finished. Gene annotation is partly from tritrypDB and partly manual. Sheet 1: Raw read counts for all ORFs. RP = ribosome profiling, FR = fragmented poly(A) + RNA. BS = bloodstream, PC = procyclic. Sheet 2: Reads for the unique gene set of genes [9], used in subsequent calculations. Sheet 3: DeSeq comparison of Jensen and Silicotryp results (total ribosome profiling reads per ORF) for procyclic forms. Sheet 4: DeSeq comparison of Jensen and Silicotryp results (total ribosome profiling reads per ORF) for bloodstream forms. Sheet 5: DeSeq comparison of Jensen and Silicotryp results (total fragmented RNA reads per ORF) for procyclic forms. Sheet 6: DeSeq comparison of Jensen and Silicotryp results (total fragmented RNA reads per ORF) for bloodstream forms. Sheet 7: ORFs that showed significant differences in ribosome profiling reads between the Jensen and Silicotryp datasets. Red are higher in Silicotryp and blue are lower. Sheet 8: Reads per million calculated for the unique gene set. Sheet 9: Alignment statistics. rp = ribosome profiling; fr = fragmented poly(A) + RNA. PC- = procyclic, BS = cultured bloodstream forms. Sheet 10: Alternative normalisation using EdgeR. (XLSX 7622 kb) Additional file 19: Table S5. Ribosome density calculations and comparisons. Sheet 1. Ribosome densities in procyclic forms. The average number of ribosomes per ORF was calculated as follows. First, we needed to correct the ribosome profiling results for the gene copy number in the genome assembly, since during the initial alignments, the reads had been spread out among all orthologues. Orthologue counts were taken from TritrypDB. Unexpected numbers were checked against our previous read alignment data [20], and in some cases manually. Read results for some gene families (such as amino acid transporters) are unreliable because related genes show different regulation. The orthologue counts used are in column D. All RPM were multiplied by the orthologue count (column H). Numbers of mRNAs per cell [16] were previously calculated assuming a total of 19,000 non-VSG mRNAs in bloodstream forms, and 43,000 non-EP-procyclin mRNAs/cell in procyclics [17]. There are about 275000 ribosomes per procyclic parasite and 125000 per bloodstream form [17]. The ribosome densities are calculated using these numbers. In practice, the densities are likely to be lower. About 5 % of ribosomes are likely to be on ORFs that were excluded from the analysis (Procyclin, VSG, ESAGs, and from repetitive elements). Approximately 70 % of all ribosomal RNA is in polysomes, 20 % is in monosomes and the rest is in subunits (our unpublished results); we do not know what proportion of the ribosomes in the monosomal fraction is engaged in translation. Sheet 2. Ribosome densities in bloodstream forms. Sheet 3. Ribosome densities and half-lives in procyclic forms, using only the “reliable” values that were chosen for modelling. Sheet 4. Ribosome densities and half-lives in bloodstream forms, using only the “reliable” values that were chosen for modelling. (XLSX 2688 kb) Additional file 20: Figure S6. Ribosome densities for mRNAs encoding proteins in different functional categories. The categories were manually assigned and can be found in the supplementary tables. The box plot indicates the median with first and third quartile, with whiskers for the 95 % confidence limits. Outliers with more than 40 ribosomes per kb were included in the calculation but are not shown. ANOVA p values were calculated after removal of impossible ribosome densities (>40/kb); the uncorrected values are indicated for classes with *p* < 0.05. (PDF 437 kb)
