# Supplementary figures and images for: Integrative analysis of the Trypanosoma brucei gene expression cascade predicts differential regulation of mRNA processing and unusual control of ribosomal protein expression
Source: BMC Genomics. 2016 Apr 26;17:306. doi: 10.1186/s12864-016-2624-3 (PMC4845500; doi:10.1186/s12864-016-2624-3)

**Model BS-A**

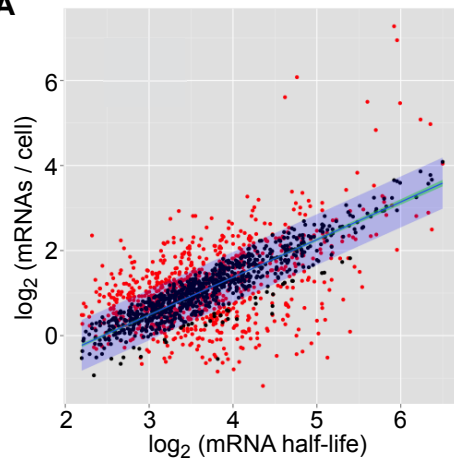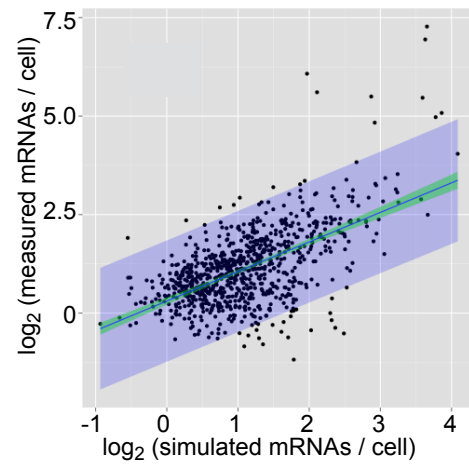

**Model BS-B**

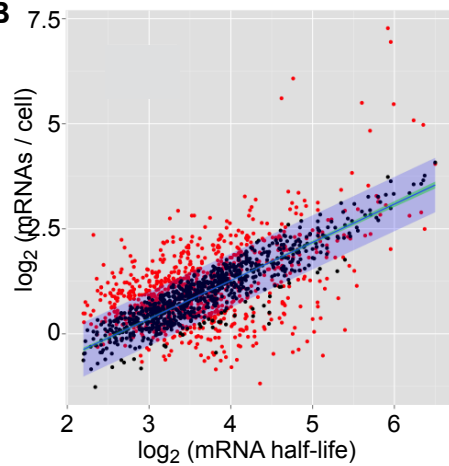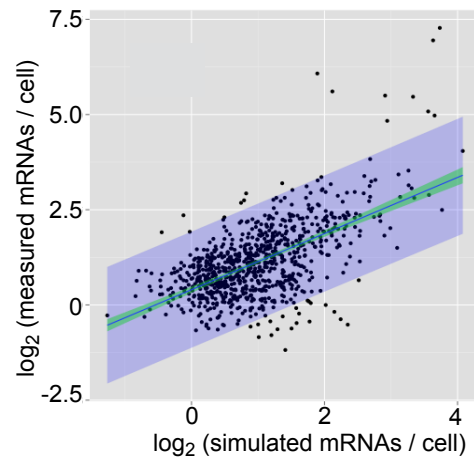

**Model BS-C**

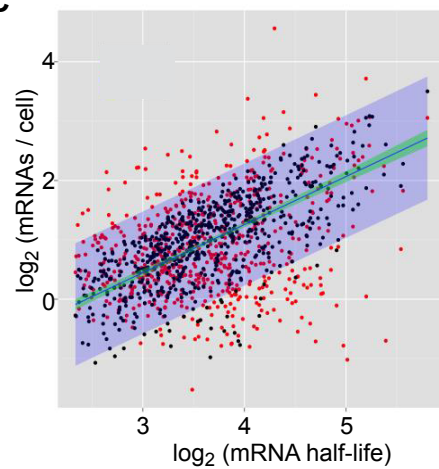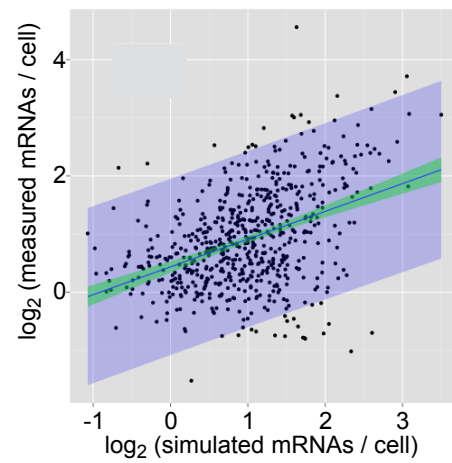

**Model BS-D**

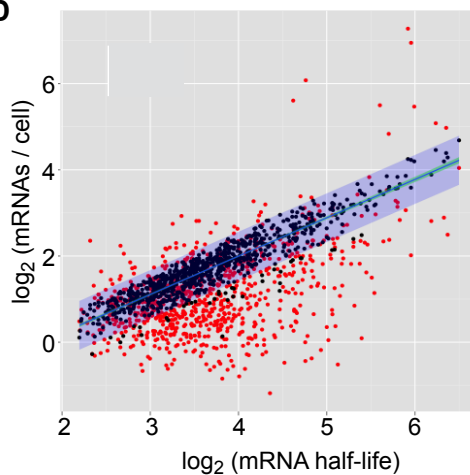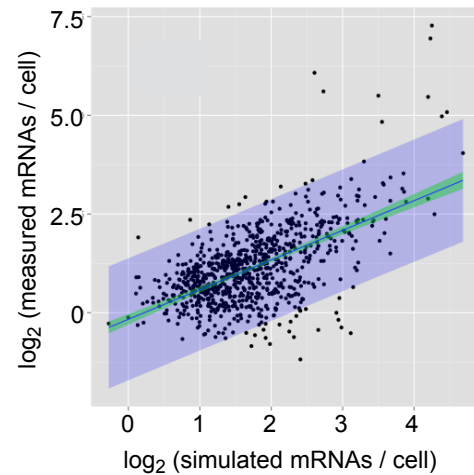

Supplement: Additional file 10: Figure S1. — Adjustments to the gene expression model for bloodstream forms. The graphs on the left show mRNA abundances plotted against their half-lives. Modelled mRNA abundances are in red, and the real mRNA abundances are in black. The green bar shows the 95 % confidence interval and blue shadowed area shows the 95 % prediction band. In the graphs on the right, the real mRNA abundances are plotted against the simulated values for the same half-life and mRNA length. The values and models used for the predictions are listed in Table 1. Log2 values were used. (PDF 1371 kb) [file 12864_2016_2624_MOESM10_ESM.pdf]

## Chosen mRNA subset

**Model BS-E**

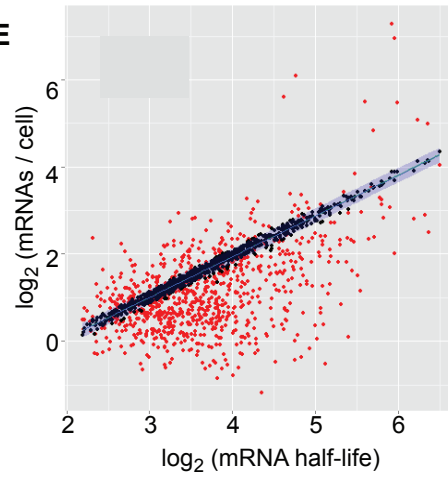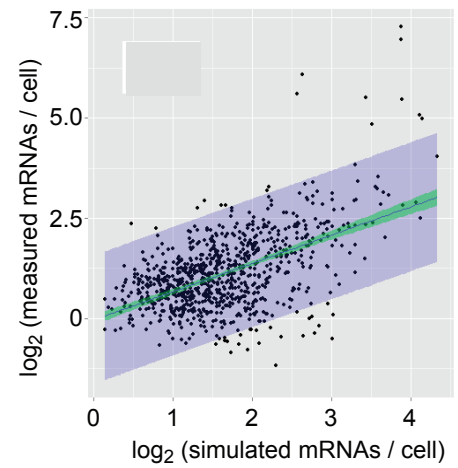

**Model BS-F**

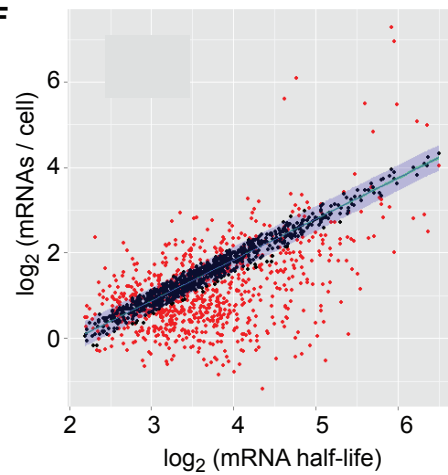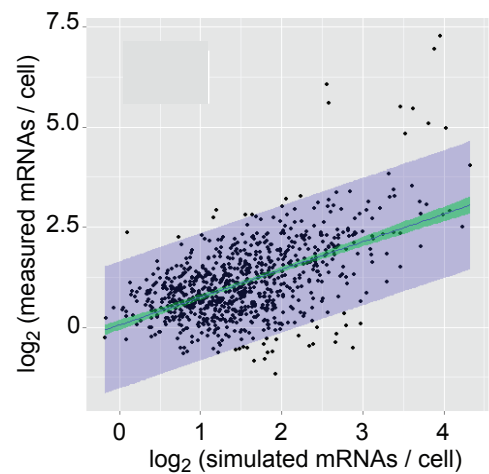

## All reliable mRNAs

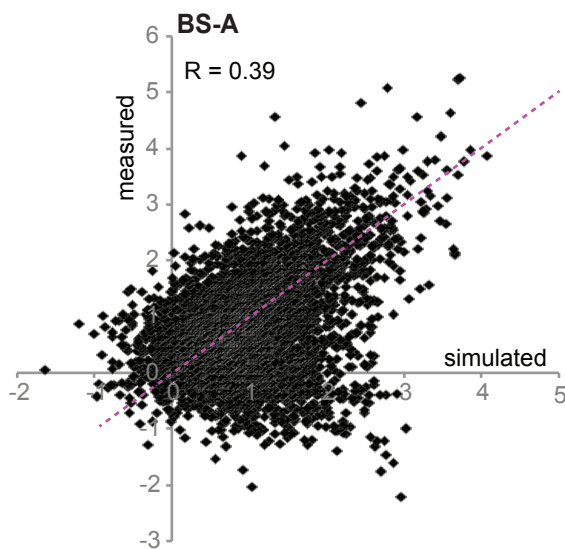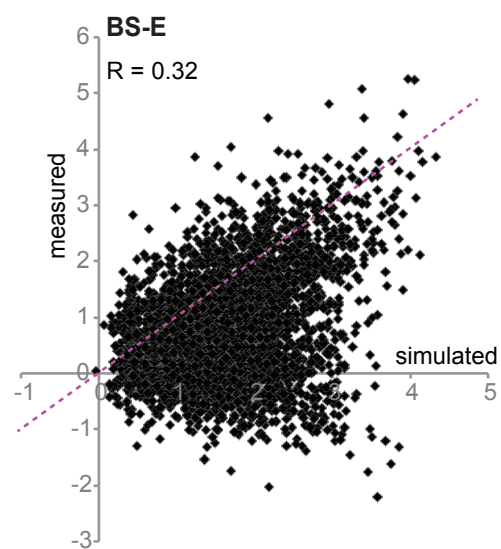

Supplement: Additional file 11: Figure S2. — Further adjustments to the gene expression model for bloodstream forms. Top four panels: The graphs on the left show mRNA abundances plotted against their half-lives. Modelled mRNA abundances are in red, and the real mRNA abundances are in black. The green bar shows the 95 % confidence interval and blue shadowed area shows the 95 % prediction band. In the graphs on the right, the real mRNA abundances are plotted against the simulated values for the same half-life and mRNA length. The values and models used for the predictions are listed in Table 1. Lower panels: These are the same as Fig. 2B, except that models BS-A and BS-E were used and log2 values are shown. (PDF 1142 kb) [file 12864_2016_2624_MOESM11_ESM.pdf]

### Model PC-A

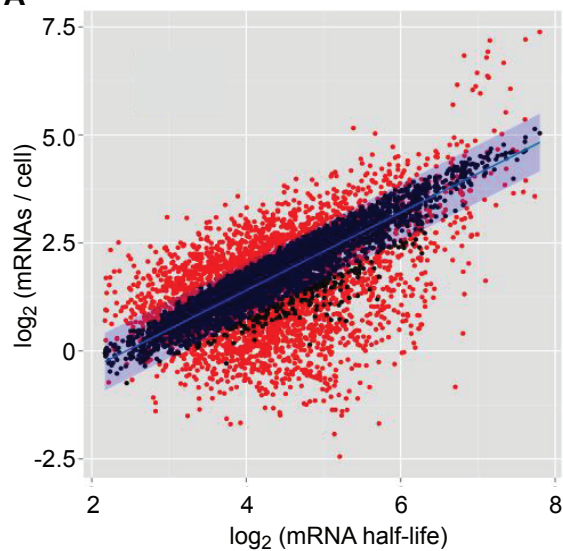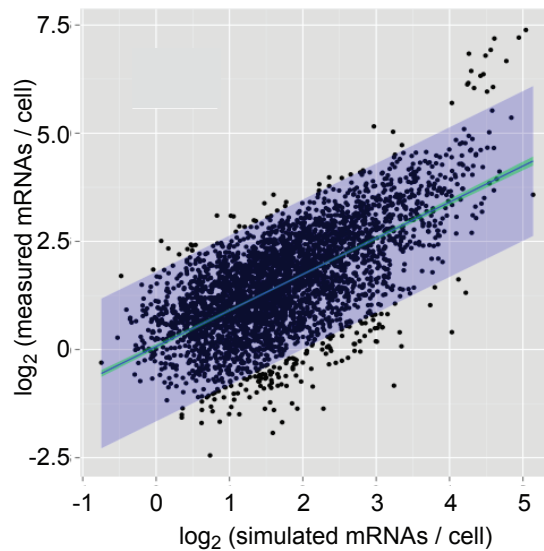

### Model PC-B

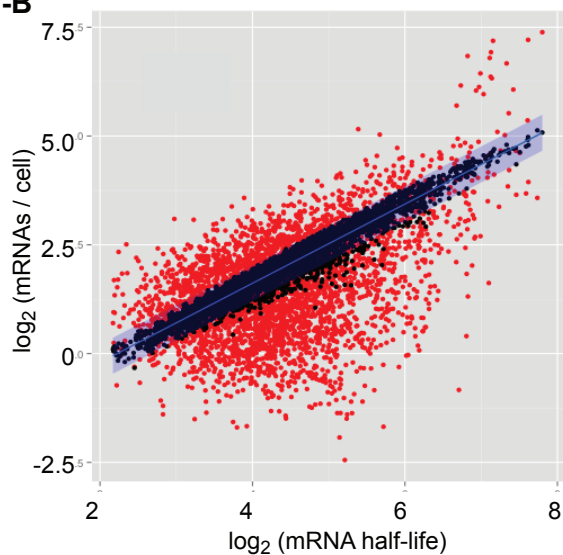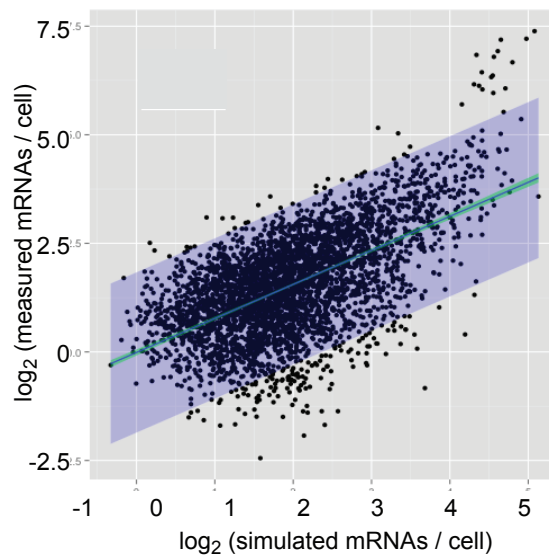

### Model PC-B

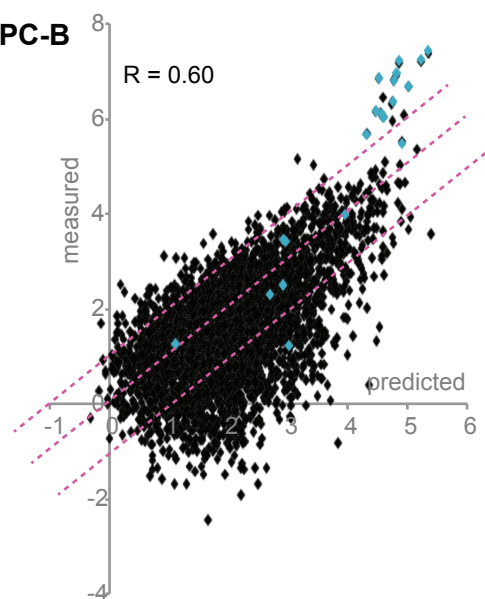

Supplement: Additional file 12: Figure S3. — Adjustments to the gene expression model for procyclic forms. Upper four panels: The graphs on the left show mRNA abundances plotted against their half-lives. Modelled mRNA abundances are in red, and the real mRNA abundances are in black. The green bar shows the 95 % confidence interval and blue shadowed area shows the 95 % prediction band. In the graphs on the right, the real mRNA abundances are plotted against the simulated values for the same half-life and mRNA length. The values and models used for the predictions are listed in Table 1. Lower panel: This is like Fig. 2a, showing the ribosomal protein mRNAs, except that the model used is PC-B and log2 values are shown (PDF 1630 kb) [file 12864_2016_2624_MOESM12_ESM.pdf]

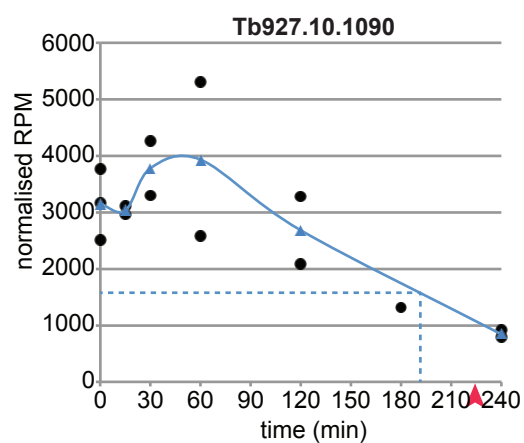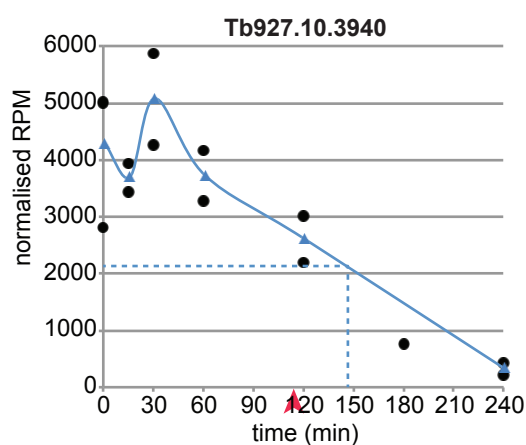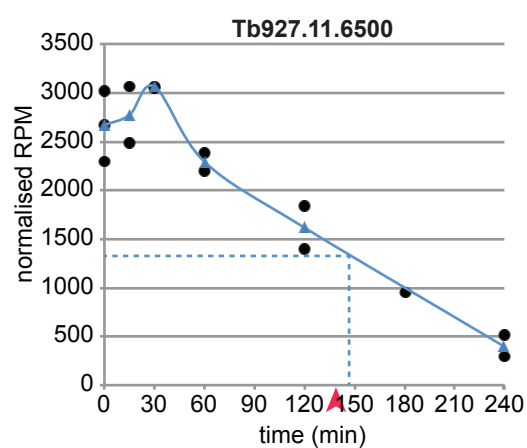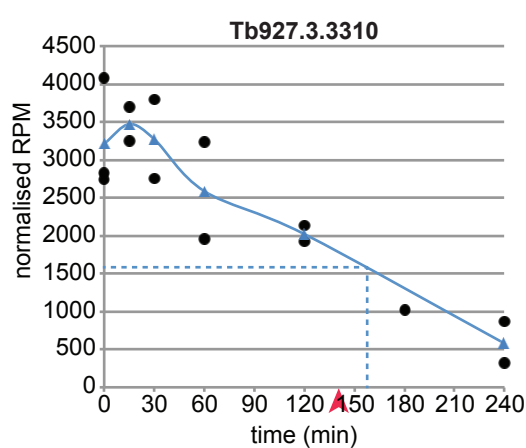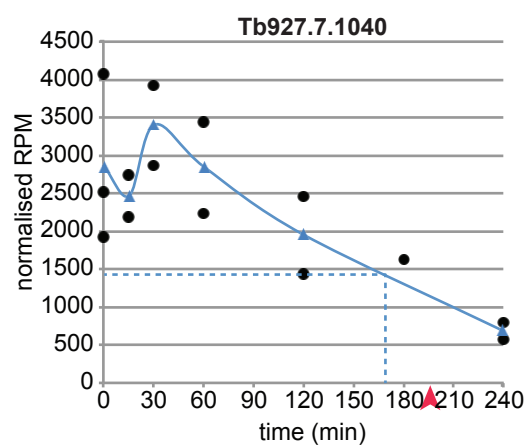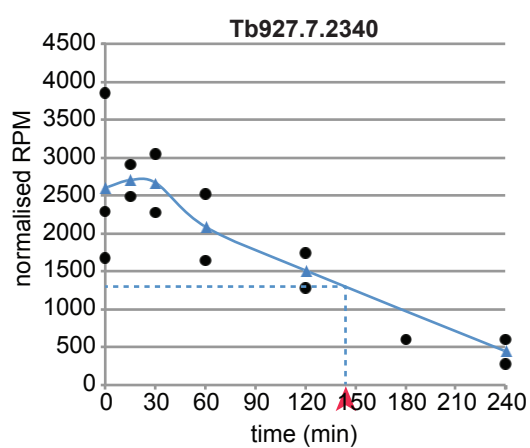

- Normalised RNASeq data
- ▲ average
- joined averages
- - - manual half-life
- ▲ calculated half-life

Supplement: Additional file 15: Figure S4. — Accuracy of automatically calculated half-lives and abundances for mRNAs encoding ribosomal proteins. The original mRNA decay results for six ribosomal protein mRNAs that were more than 4 times more abundant than expected in procyclic forms are shown, as reads per million reads corrected for total mRNA abundance [16]. The key is on the Figure. The red arrow indicates the half-life that was calculated in [16] according to [57]. (PDF 423 kb) [file 12864_2016_2624_MOESM15_ESM.pdf]

RNA/cell/gene: Observed/predicted

0 1 2 3 4 5 6

Procyclic forms

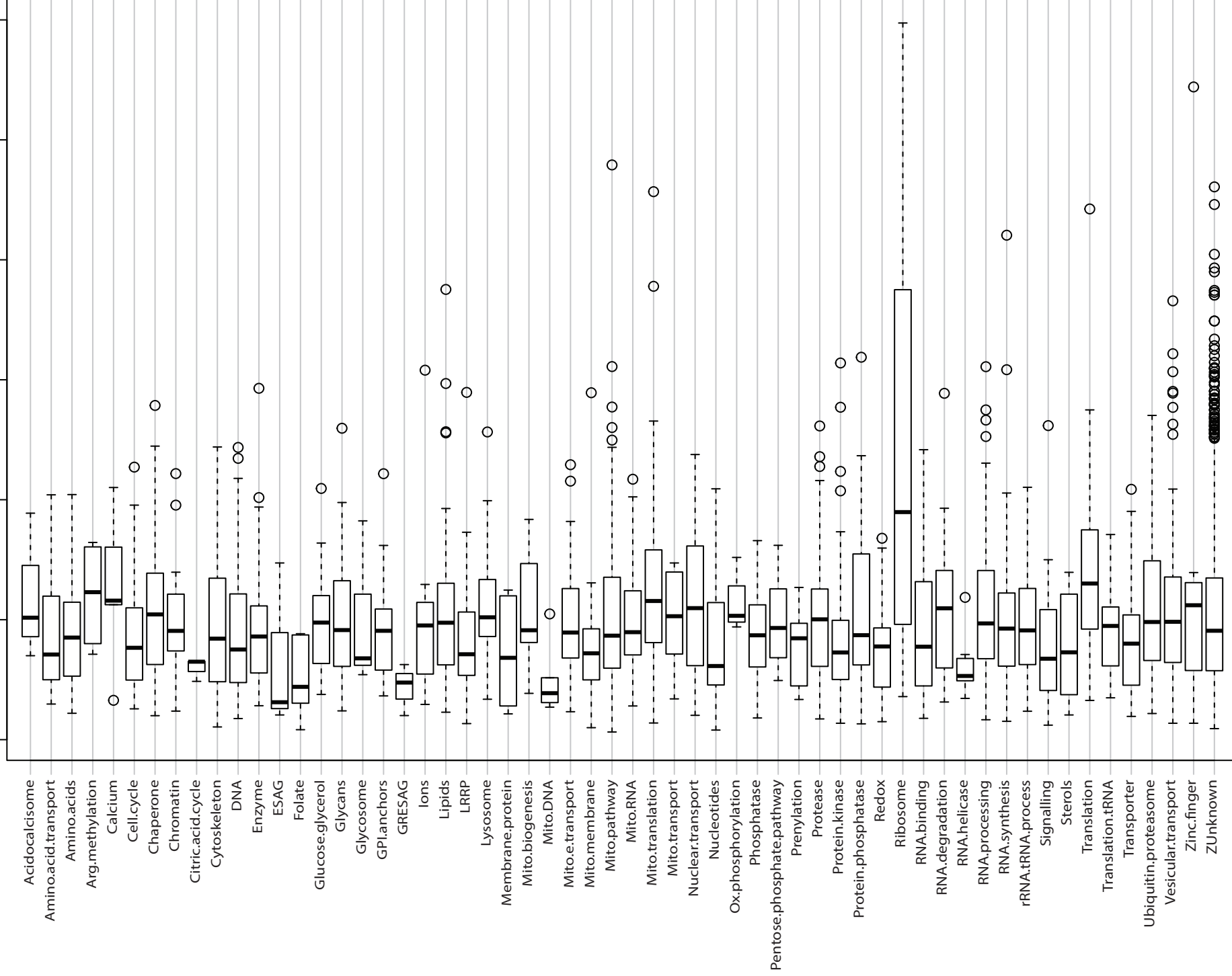

Supplement: Additional file 16: Figure S5. — Ratio between observed and predicted mRNA abundances in procyclic forms, for mRNAs encoding proteins in different functional categories. The categories were manually assigned and can be found in the supplementary tables. The box plot indicates the median with first and third quartile, with whiskers for the 95 % confidence limits. The ANOVA p-value for ribosomal protein mRNAs was 0.001. (PDF 395 kb) [file 12864_2016_2624_MOESM16_ESM.pdf]

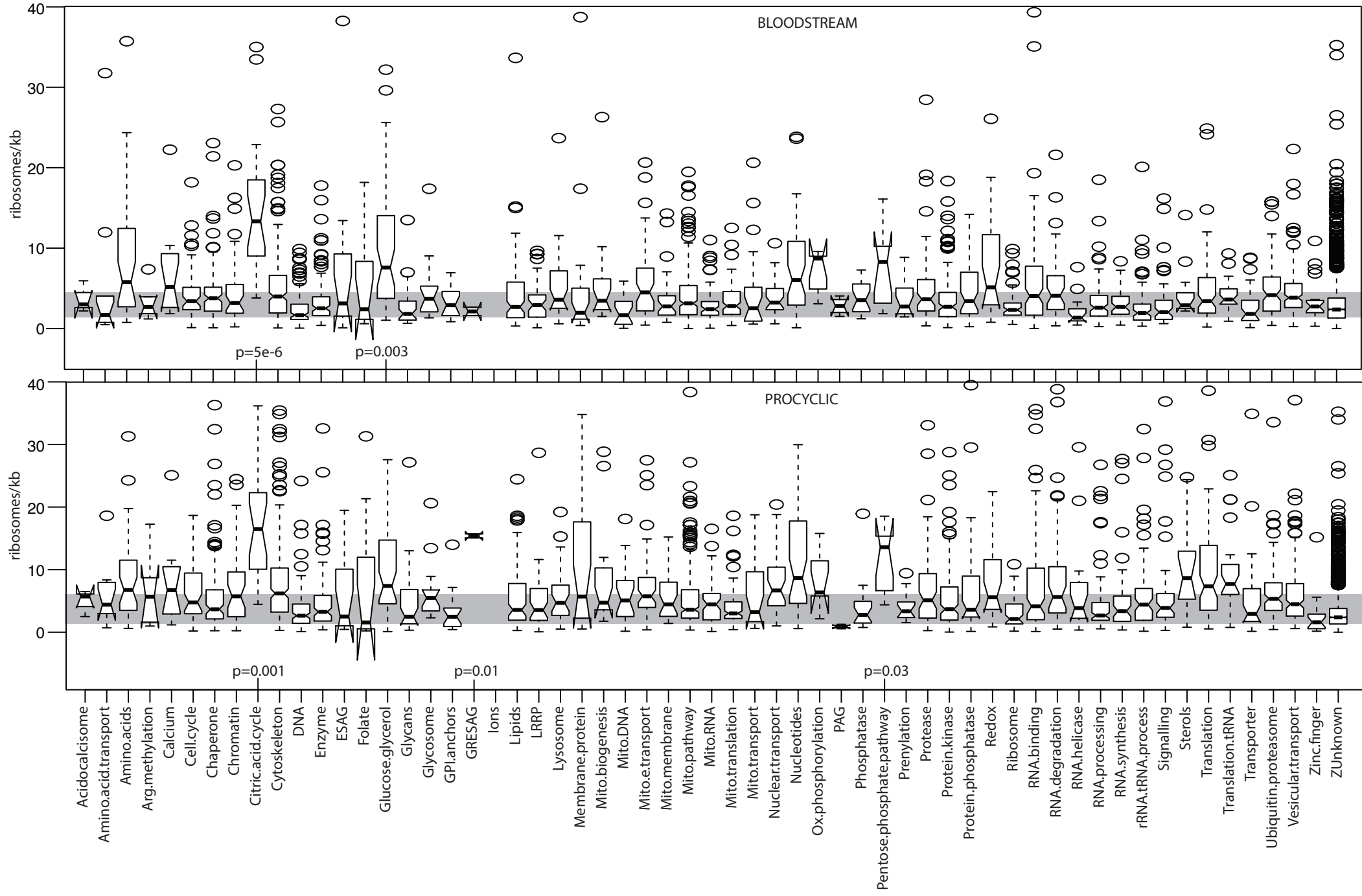

Supplement: Additional file 20: Figure S6. — Ribosome densities for mRNAs encoding proteins in different functional categories. The categories were manually assigned and can be found in the supplementary tables. The box plot indicates the median with first and third quartile, with whiskers for the 95 % confidence limits. Outliers with more than 40 ribosomes per kb were included in the calculation but are not shown. ANOVA p values were calculated after removal of impossible ribosome densities (>40/kb); the uncorrected values are indicated for classes with p < 0.05. (PDF 437 kb) [file 12864_2016_2624_MOESM20_ESM.pdf]
